# Comparative Safety and Efficacy of Remdesivir Versus Remdesivir Plus Convalescent Plasma Therapy (CPT) and the Effect of Timing of Initiation of Remdesivir in COVID-19 Patients: An Observational Study From North East India

**DOI:** 10.7759/cureus.19976

**Published:** 2021-11-28

**Authors:** Dibya J Sharma, Aparajita Deb, Phulen Sarma, Bipadabhanjan Mallick, Prithwiraj Bhattacharjee

**Affiliations:** 1 Internal Medicine and Gastroenterology, Silchar Medical College and Hospital, Silchar, IND; 2 Internal Medicine, Silchar Medical College and Hospital, Silchar, IND; 3 Pharmacology, Post Graduate Institute of Medical Education and Research, Chandigarh, IND; 4 Gastroenterology, Kalinga Institute of Medical Sciences, Bhubaneswar, IND

**Keywords:** ventilation, icu, spo2, follow up, ferritin, outcome, mortality, convalescent plasma therapy, remdesivir, covid-19

## Abstract

Introduction

As per the COVID-19 treatment guidelines of India, remdesivir and convalescent plasma therapy (CPT) are indicated in moderate and severe patients. In this study, we have evaluated the comparative safety and efficacy of remdesivir versus remdesivir CPT combination and effect of early versus late initiation of remdesivir.

Materials and methods

A hospital-based observational study was conducted among hospitalized moderate and severe COVID-19 patients treated with either remdesivir and/or CPT as per national guidelines. Response to therapy was evaluated in terms of mortality, mechanical ventilation requirement, ICU requirement, and safety.

Results and observations

A total of 95 moderate and severe COVID-19 patients on remdesivir (n=35) or remdesivir + CPT combination (n=60) were included. Both the remdesivir and remdesivir + CPT groups were comparable in terms of baseline characteristics, however, proportion of patients with baseline serum creatinine >1.5 was higher in the remdesivir group. No difference was seen between both the groups in terms of mortality, mechanical ventilation requirement, ICU requirement, and safety parameters in the overall moderate and severe COVID-19 populations and when each of these severity categories (moderate and severe) were analyzed separately. Early initiation (<9 days from symptom onset) of remdesivir was associated with better treatment outcome in terms of mortality and requirement of ICU. Post-therapy shortness of breath and LFTs (liver function tests) elevation was more in the late initiation of remdesivir group, which may be due to the lack of efficacy and subsequent disease progression or a direct effect of the drug. The beneficial effect of remdesivir was maintained even after adjustment for important prognostic factors and baseline imbalances (age, sex, disease severity, CPT use, and serum creatinine level).

Conclusions

Early initiation of remdesivir was associated with clinical benefit in terms of mortality and mechanical ventilation requirement. However, addition of convalescent plasma therapy as an additional therapeutic modality to remdesivir was not found to be beneficial.

## Introduction

Coronavirus disease 2019 (COVID-19), which is caused by severe acute respiratory syndrome coronavirus 2 (SARS-CoV-2), had its first case reported in Wuhan, China in December 2019 [1]. On January 30, 2020, the World Health Organization declared that the SARS-CoV-2 epidemic was a public health emergency of international concern [2]. Its epidemiology, transmission dynamic, clinical outcomes, and case fatality rates show considerable variation among various ethnic groups.

The common clinical features of COVID-19 are fever, diarrhea, cough, fatigue, muscle soreness, rhinorrhea, ageusia, anosmia, sore throat, and respiratory distress. However, other atypical presentations like conjunctivitis, rheumatologic manifestations, etc., are not uncommon [3,4]. Clinically, it has been classified as mild, moderate, or severe as per oxygen saturation and the presence of organ failure [5]. Radiologically, abnormal chest X-rays or computed tomography (CT) scans can predict the severity and extent of lung involvement [6]. Lymphopenia, leucopenia, thrombocytopenia, elevated inflammatory markers (e.g., C-reactive protein, serum ferritin level), abnormal liver and renal function, elevation of cardiac biomarkers, and decreased albumin are ancillary parameters detected during laboratory evaluation in patients with COVID-19 infection, while positive real-time polymerase chain reaction (RT-PCR) from nasopharyngeal swab remains the gold standard test for diagnosis [7].

The mild patients can be managed by the treatment of clinical symptoms with supportive therapy, while moderate and severe cases require supplemental oxygen, immunomodulatory, and investigational therapy. Some of the severe patients who deteriorate on oxygen supplementation need mechanical ventilatory support.

Presently used therapeutic agents for the management of COVID-19 include steroid therapy [8]; remdesivir [9]; chloroquine; hydroxychloroquine [10-12]; favipiravir [13]; ivermectin [14]; convalescent plasma therapy (CPT) [15]; and adjunctive agents including zinc, vitamin D [16], folic acid [17], and anticoagulants [18].

Remdesivir is an injectable antiviral drug that inhibits SARS-CoV-2 RNA-dependent RNA polymerase, which is vital for viral RNA synthesis [1]. In in-vitro conditions, remdesivir inhibited SARS-CoV-2 [19]. Remdesivir was granted emergency use authorization by the United States Food and Drug Administration (US FDA) on October 2020 for use in hospitalized patients with severe COVID-19 [20].

Convalescent plasma therapy is an investigational therapy where plasma from recovered COVID-19 patients is infused into compatible critically ill COVID-19 patients. The US FDA has granted emergency use authorization to CPT for hospitalized COVID-19 patients in August 2020 [21].

Many studies have reported the safety and efficacy of remdesivir, e.g., remdesivir versus placebo [22,23], remdesivir versus standard of care [24,25], remdesivir + baricitinib versus remdesivir + placebo [26], and remdesivir 5 days versus 10 days [27], however, comparison between remdesivir and remdesivir + CPT for COVID-19 treatment is inconclusive as some studies have shown benefit while others did not observe any improvement. However, the early initiation of remdesivir versus late initiation was compared previously by one dedicated study in a peer reviewed database [28].

The Indian standard treatment for COVID-19 infection has been frequently revised and patients have been categorized into mild, moderate, and severe cases based on respiratory distress, respiratory rate, and oxygen saturation [29]. The guidelines have constantly changed [29,30] with the availability of more evidence regarding the natural course of the disease and the behaviour of the virus [5]. The management of patients with moderate and severe illness includes respiratory support to maintain the optimal target for oxygen saturation (SpO2), anti-inflammatory therapy (steroids), anti-coagulation (low molecular weight heparin, unless there is no contraindication or high risk of bleeding) and intensive monitoring. Remdesivir injection was indicated in patients with moderate and severe illness who required supplemental oxygen or mechanical ventilation if not contraindicated by hyper-transaminesemia (aspartate aminotransferase [AST] or alanine aminotransferase [ALT] more than five times upper limit of normal), estimated glomerular filtration rate (eGFR) <30 ml/min, pregnancy, lactation, and allergy to remdesivir. CPT has been indicated in compatible moderate and severe cases with increasing oxygen requirements despite the use of steroids without immunoglobulin allergy [29].

The current observational study was undertaken in the Silchar Medical College and Hospital, the only tertiary care hospital in southern Assam located in North East India, which has a unique ethnic and genetic background in comparison to the rest of India. Here, we compared the safety and efficacy data of patients on two major treatment modalities (remdesivir and remdesivir + CPT) for patients with moderate and severe COVID-19 as per standard treatment guidelines. With the ever-increasing mutation of the virus, it has become all the more essential to know the natural course of the virus among different races, ethnicities, and geographical locations.

## Materials and methods

This study was conducted in the COVID-19 ward and COVID-19 ICU of the Silchar Medical College and Hospital, in Silchar, Assam, and the study was approved by the institutional ethics committee of Silchar Medical College by letter number SMC/754 (IRB - SMC/754). The trial is registered with the Clinical Trials Registry of India.

Primary objective

The primary objective was to compare the efficacy (in terms of mortality, mechanical ventilation, and ICU requirement) and safety of remdesivir versus remdesivir + CPT among moderate and severe COVID-19 patients.

Secondary objective

The secondary objective was to compare the efficacy (in terms of mortality, mechanical ventilation and ICU requirement) and safety of early remdesivir (within 9 days of symptom onset) versus late remdesivir (>9 days from symptom onset) initiation.

Inclusion criteria

The inclusion criteria consisted of the following patient characteristics:

Patients who tested positive for COVID-19 by RT-PCR or rapid antigen test (RAT) and hospitalized as a moderate or severe case as well as who have received either CPT and/or remdesivir injection.

Exclusion criteria

The exclusion criteria consisted of the following patient characteristics:

Patients who did not provide consent for the study, pregnant or lactating women, and patients who already have chronic kidney disease, malignancy, or psychiatric illness that can adversely impact the course of the disease, children younger than 12 years of age, and pneumonia other than COVID-19.

Definitions

The following definitions of COVID-19 severity were used based on the national guidelines issued by the Ministry of Health, Government of India [5].

Mild COVID-19: Patients with uncomplicated mild symptoms without any evidence of hypoxia (SpO2 ≥95% on room air) or breathlessness [5].

Moderate COVID-19: Adolescent or adult symptomatic patients with hypoxia (SpO2 <94% on room air, range 90%-94%) and/or dyspnea and respiratory failure (indicated by respiratory rate ≥24 breaths/minute) [5].

Severe COVID-19: Adolescent or adult symptomatic patients with clinical signs of pneumonia and SpO2 <90% on room air or respiratory rate >30 breaths/min (any of the last two criteria) [5].

Patient treatment

All patients were treated as per standard treatment guidelines [5] in the COVID-19 care center. As per standard guidelines, remdesivir was administered to patients older than 12 years (indicated for both sexes, but among females indicated only for non-pregnant or non-lactating individuals) requiring supplemental oxygen with AST and or ALT <5 times the ULN (upper limit of normal), without severe renal impairment (eGFR <30 ml/min/m2 or need for hemodialysis) [5]. CPT was considered among patients with a progressively increasing oxygen requirement despite the use of steroids [5].

Remdesivir Dose Details

Remdesivir was given as a 200-mg injection diluted in 100 ml normal saline. It was infused over 60-90 minutes on day 1, followed by a 100 mg injection in 100 ml normal saline for the next four days [5].

Plasma Therapy Details

Only ABO compatible and cross-matched donor plasma was used for therapy (donor plasma was provided by recovered COVID-19 patients resolution of symptoms at a minimum of 28 days prior to donation) with a plasma immunoglobulin G (IgG) titer above 1:640 (against receptor-binding domain [RBD] of s- protein) and normal complete blood count. Recipients were monitored for 24 hours post transfusion for any transfusion-related adverse events [5].

Dose of CPT

A single 200 ml dose was administered intravenously slowly over 2 hours. A second dose (preferably from a different donor) was transfused among non-responders after 24 hours, as per guidelines. However, CPT therapy was subjected to availability and matching. In the absence of matching and suitable donor plasma, patients were given remdesivir or other therapies alone as per standard guidelines [5,29]. 

Routine Monitoring and Clinical Care

A detailed clinical history was obtained and a complete physical examination was performed in all cases. All routine hematological and biochemical investigations, such as complete blood count, renal and liver function tests, and evaluations of inflammatory markers, such as serum ferritin and lactate dehydrogenase (LDH) were determined. The necessary radiological investigations were carried out including chest X-ray and high-resolution computed tomography of the chest. Data were entered into a case-record form and patients were categorized as mild, moderate, or severe disease according to the SpO2 and respiratory rate documented on presentation and as per the national and state guidelines. Comorbid conditions, if present, were also recorded.

Study design

The present study was a hospital-based non-randomized prospective observational study. Moderate and severe COVID-19 patients were treated as per standard treatment guidelines by hospital physicians. Treatment and outcome data were recorded and analyzed.

Follow-up

All patients were followed up till their discharge or the time of death. Survivors were called for a follow-up visit 14 days after the date of discharge, and the SpO2 was recorded to assess the improvements in arterial oxygen saturation.

Evaluation of efficacy and safety parameters and data collection

All patients were subjected to routine monitoring. All parameters including clinical and laboratory outcomes were recorded in a case record form. Following treatment initiation, all patients were rigorously monitored for clinical and laboratory evidence (of any signs of toxicity by liver function tests [LFTs] and complete hemogram).

Sample size

All the patients suffering from moderate and severe COVID-19 admitted to the COVID-19 ward and COVID-19 ICU of the Silchar Medical College and Hospital over a period of four months were included in the analysis.

Statistical analysis

Data was entered into Microsoft Excel (Microsoft Corporation, Redmond, USA) and statistical analysis was carried out using SPSS software (IBM Corp., Armonk, USA). Normally distributed quantitative data was presented as mean ± standard deviation (S.D.), and non-normally distributed data was represented as median (interquartile range, IQR). For hypothesis testing, an independent students’ t-test or Mann-Whitney U test was applied depending on the distribution of dependent quantitative data. In cases where the association between two qualitative parameters was evaluated, data was presented as proportions and the Chi-squared test or Fischer’s Exact test were performed for hypothesis testing. Safety and efficacy parameters were further adjusted with important prognostic factors and baseline imbalances. A p-value ≤0.05 was considered statistically significant.

Ethical considerations

The study obtained approval (letter no. SMC/754) from the ethical committee of our institute and was conducted as per guidelines of the Declaration of Helsinki. Informed consent was obtained from the patients or their attendants before inclusion in our study. 

## Results

Participant screening

**Figure 1:**
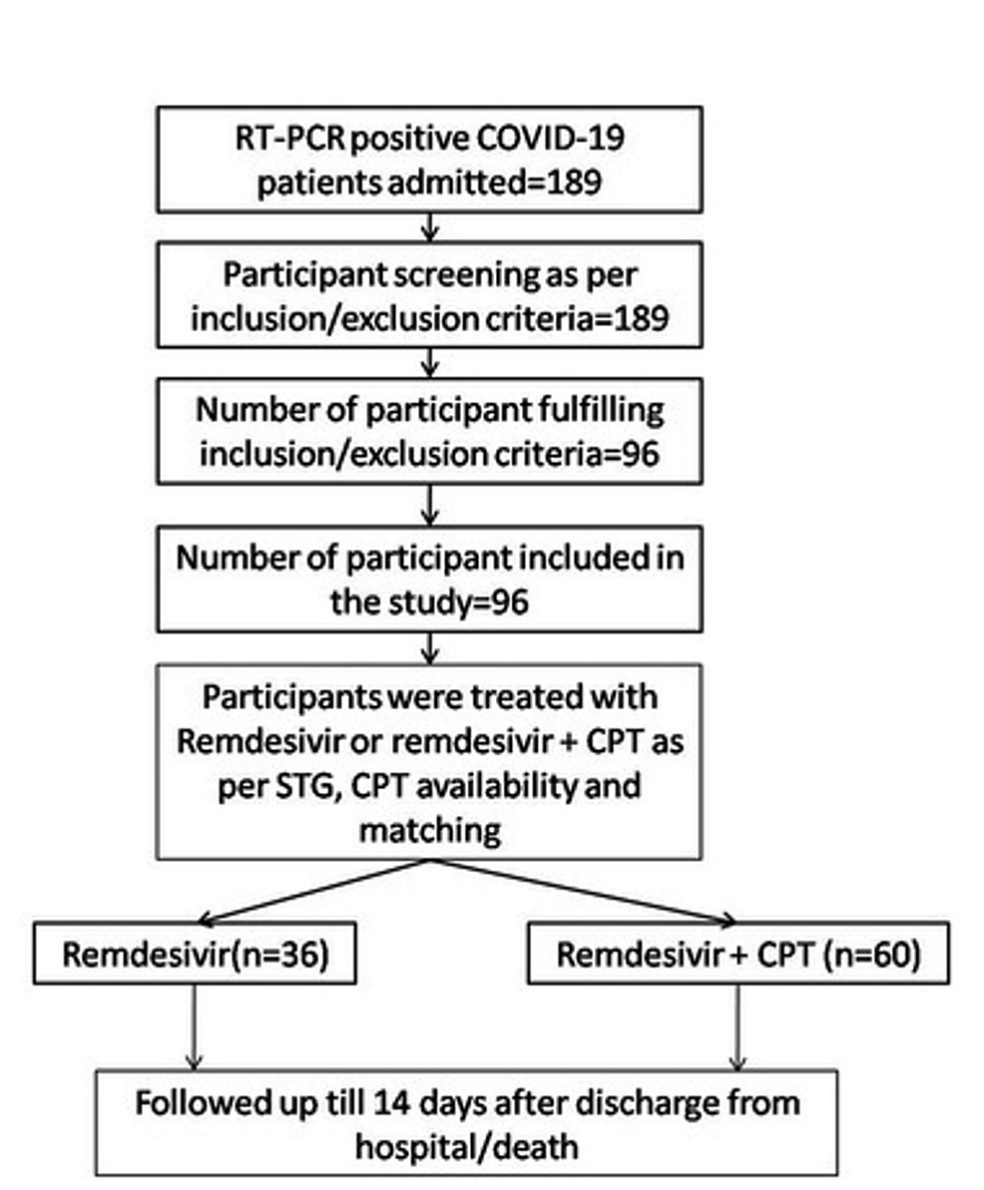
Remdesivir versus remdesivir + CPT RT-PCR: Reverse transcription polymerase chain reaction, CPT: convalescent plasma therapy; STG: standard treatment guidelines

A total of 189 patients were found to be COVID-19 positive. Among them, 96 patients suffering from moderate (n=61) and severe (n=35) COVID-19 were included in our study. CPT was non-matching in many cases in a random manner, although it was indicated as per national treatment guidelines. However, remdesivir was given in all cases. Ultimately, 36 patients received remdesivir alone, while 60 patients received both (remdesivir and CPT) (Figure 1).

Remdesivir versus remdesivir plus CPT

Both treatment groups (remdesivir and remdesivir + CPT) were comparable in terms of age, sex distribution, SpO2 at baseline, respiratory rate at baseline, pulse rate, total leukocyte count (TLC), proportion of patients with serum ferritin level>1000, AST, ALT, proportion of patients with LDH>250, Glasgow coma scale (GCS), distribution of different comorbidities (the comorbidities present were diabetes and hypertension), distribution of different severity categories of patients (moderate and severe COVID-19), and other co-interventions prescribed. The common co-medications prescribed were low molecular weight heparin (LMWH) and dexamethasone, which was prescribed in 100% cases in both treatment groups. Other co-medications prescribed were hydroxychloroquine (HCQ), azithromycin, and favipiravir. However, no difference was noted in the prescription of these co-medications between the two groups and the population with a serum creatinine level >1.5 at baseline was higher in the remdesivir group compared to that in the remdesivir + CPT group (p=0.025). The different presenting symptoms in both the groups were fever, sore throat, shortness of breath (SOB), cough, diarrhea, nausea, vomiting, etc., which were similar in both the groups (Table 1).

**Table 1:** Baseline characteristics of patients on remdesivir alone and remdesivir + convalescent plasma therapy SpO2: saturation of oxygen; TLC: total leucocyte count; HTN: hypertension; BUN: blood urea nitrogen; AST: aspartate aminotransferase; ALT: alanine aminotransferase; ULN: upper limit of normal; LDH: lactate dehydrogenase; CRP: C-reactive protein; GCS: Glasgow coma scale; IQR: interquartile range; HCQ: hydroxychloroquine; LMWH: low molecular weight heparin; SOB: shortness of breath.

Parameter		Remdesivir (n=36)	Remdesivir + CPT (n=60)	P value
Age, years [mean ± S.D.]		48 ± 12	51 ±14	0.290
% Male		24 (66.7%)	45 (75%)	0.498
SpO_2_ at baseline [mean ± S.D.]		87 ± 9	88.9 ± 5	0.265
SpO_2_ at baseline	<90	12 (33.3%)	23 (38.3%)	0.622
	90-94	24 (66.7%)	37 (61.67%)	
Respiratory rate [mean ± S.D.]		26.6 ± 3	26.6 ± 4	0.928
Pulse rate >100		26 (72.22%)	49 (81.67%)	0.395
TLC [Median (IQR)]		7890 (6070)	7800 (4305)	0.527
TLC	<4000	6 (16.67%)	10 (16.67%)	0.131
	4000-11000	17 (47.22%)	39 (65%)	
	>11000	13 (36.11%)	11 (18.33%)	
Serum ferritin >1000		23 (63.89%)	41 68.33%)	0.793
Serum creatinine >1.5		7 (19.44%)	3 (5%)	0.025
Serum creatinine		1.17± 0.45	1 ± 0.3	0.036
BUN>20		17 (47.22%)	18 (30%)	0.07
ALT>2 ULN		20 (55.55%)	28 (46.67%)	0.325
AST>2 ULN		20 (55.55%)	27 (45%)	0.254
Pro-calcitonin		0.729 ± 0.74	0.712 ± 0.96	0.938
LDH >250		34 (94.44%)	55 (91.67%)	0.29
CRP		63.8 ± 32	74.1 ± 34	0.178
D-Dimer [Median (IQR)]		1.5 (1.1)	1.6 (1)	0.143
GCS	<8	1 (2.78%)	1 (16.67%)	0.927
	8-12	5 (13.89%)	9 (15%)	
	>12	30 (83.33%)	50 (83.33%)	
Comorbidities	Diabetes	20 (55.55%)	36 (60%)	0.785
	HTN	15 (41.67%)	27 (45%)	0.839
Severity at presentation	Moderate	24 (66.78%)	37 (61.67%)	0.622
	Severe	12 (33.33%)	23 (38.33%)	
Other co-medications	HCQ	35 (97.22%)	55 (91.67%)	0.88
	Azithromycin	32 (88.89%)	60 (100%)	0.87
	Fevipiravir	3 (83.33%)	5 (8.33%)	1.00
	LMWH	36 (100%)	60 (100%)	NA
	Dexamethasone	36 (100%)	60 (100%)	NA
Clinical symptoms	Vomiting	2 (5.56%)	5 (8.33%)	0.637
	Nausea	14 (38.89%)	23 (38.33%)	0.872
	Diarrhea	8 (22.22%)	31 (51.67%)	0.006
	SOB	25 (69.44%)	38 (63.33%)	0.421
	Cough	28 (77.78%)	42 (70%)	0.286
	Sore throat	18 (50%)	33 (55%)	0.736
	Fever	28 (77.78%)	41 (68.33%)	0.219

Comparative safety and efficacy of Remdesivir versus Remdesivir plus CPT

In the overall moderate and severe COVID-19 population, no difference was observed in any of the efficacy (mortality, and mechanical ventilation and ICU requirement) and safety parameters. When the severity categories of the population were analyzed separately (separate analysis of patients with moderate and severe COVID-19), there were no statistically significant differences between the two groups in any of the efficacy and safety parameters. The most common adverse events observed were nausea, post-therapy worsening of dyspnea and a rise in liver enzyme expression (ALT) (Table 2).

**Table 2:** Comparative safety and efficacy of remdesivir versus remdesivir + CPT in patients with COVID-19 MV: mechanical ventilation; ALT: alanine aminotransferase; CPT: convalescent plasma therapy

		All Moderate + Severe cases (n=96)			Moderate cases (n=61)			Severe cases (n=35)		
Endpoint		R (n=36)	R + CPT (n=60)	P value	R (n=24)	R + CPT (n=37)	P value	R (n=12)	R + CPT (n=23)	P value
Mortality		2 (5.55%)	8 (13.33%)	0.243	1 (4.17%)	3 (8.1%)	0.570	1 (8.33%)	5 (21.73%)	0.318
Requirement of MV		2 (5.55%)	10 (16.67%)	0.111	1 (4.17%)	5 (13.51%)	0.231	1 (8.33%)	5 (21.73%)	0.318
Requirement of ICU		13 (36.11%)	34 (56.67%)	0.051	10 (41.67%)	22 (59.45%)	0.174	3 (25%)	12 (52.17%)	0.123
Post therapy side effect	Nausea	16 (44.44%)	34 (56.67%)	0.302	14 (58.33%)	26 (43.33%)	0.453	2 (16.67%)	8 (34.78%)	0.260
	Worsening dyspnea	8 (22.22%)	8 (13.33%)	0.258	5 (20.83%)	3 (5%)	0.150	3 (25%)	5 (21.73%)	0.827
	High ALT	2 (5.55%)	7 (11.67%)	0.320	1 (4.17%)	4 (10.81%)	0.355	1 (8.33%)	3 (13.04%)	0.687

Baseline characteristics: remdesivir initiation within 9 days (early initiation) versus initiation >9 days (late initiation) from the onset of symptoms

Among patients receiving remdesivir, 68 received the drug within 9 days of the onset of their first symptom (early initiation group) and 27 patients received 9 days after the onset of their first symptom (late initiation group). Both treatment groups were comparable in terms of demographic characteristics, including age and sex distribution, and in their SpO2 at baseline, respiratory rate at baseline, pulse rate, TLC, proportion of patients with serum ferritin level >1000, AST, ALT, proportion of patients with LDH>250, GCS, distribution of different co-morbidities (diabetes and hypertension were identified), distribution of different severity categories of patients (moderate and severe COVID-19), and other co-interventions prescribed. Both groups were comparable in terms of co-medications used. The proportion of patients with a serum creatinine level >1.5 was higher in the remdesivir early initiation group (p=0.031). The baseline characteristics are depicted in Table 3.

**Table 3:** Baseline characteristics: remdesivir initiation within 9 days (early initiation) versus initiation >9 (late initiation) days from symptom onset SpO2: saturation of oxygen; TLC: total leucocyte count; HTN: hypertension; BUN: blood urea nitrogen; AST: aspartate aminotransferase; ALT: alanine aminotransferase; ULN: upper limit of normal; LDH: lactate dehydrogenase; GCS: Glasgow coma scale; IQR: interquartile range; SOB: shortness of breath; RBS:

Parameter		Remdesivir within 9 days of symptom onset (n=68)	Remdesivir initiated >9 days from symptom onset (n=27)	P value
Age, years [mean ± S.D.]		52± 12	50 ± 14	0.539
% Male [n(%)]		47 (69.11%)	22 (81.48%)	0.460
SpO_2_ at baseline [mean ± S.D.]		87.5 ± 6	88 ± 7	0.606
SpO_2_ at baseline	<90 [n(%)]	24 (35.29%)	11 (40.78%)	0.400
	90-94 [n(%)]	44 (64.7%)	16 (59.25%)	
Respiratory rate [mean ± S.D.]		26± 3	27±4	0.298
Pulse rate [mean ± S.D.]		106 ± 9	103 ± 11	0.241
Pulse rate >100 [n(%)]		49 (78.05%)	26 (96.29%)	0.040
TLC [Median (IQR)]		7873 (5470)	6744± 5125	0.367
TLC	<4000 [n(%)]	11 (16.17%)	5 (18.51%)	0.700
	4000 - 11000 [n(%)]	40 (58.82%)	16 (59.25%)	
	>11000 [n(%)]	17 (25%)	6 (22.22%)	
Serum ferritin >1000 [n(%)]		45 (66.17%)	18 (66.67%)	0.713
Serum creatinine >1.5 [n(%)]		10 (14.7%)	0	0.031
Serum creatinine [mean ± S.D.]		0.99±0.22	1.09 ± 0.42	0.198
BUN>20 [n(%)]		27 (39.7%)	7 (25.92%)	0.142
ALT>2 ULN [n(%)]		33 (48.52%)	14 (51.85%)	1.000
AST>2 ULN [n(%)]		32 (47.05%)	14 (51.85%)	0.893
S. Procalcitonin [Median (IQR)]		0.4 (1.4)	0.3 (0.6)	0.66
LDH >250 [n(%)]		61 (89.7%)	27 (100%)	0.468
CRP [Median (IQR)]		72 (70.5)	102 (62)	0.029
D-Dimer [Median (IQR)]		1.5 (0.6)	1.8 (1.5)	0.005
RBS [mean ± S.D.]		207 ± 71	189 ± 67	0.239
GCS [mean ± S.D.]		13.8 ± 2	14.4±1.37	0.223
GCS	<8 [n(%)]	0	1 (3.7%)	0.170
	8-12 [n(%)]	11 (16.17%)	4 (14.81%)	
	>12 [n(%)]	57 (83.82%)	22 (81.48%)	
Comorbidities	Diabetes [n(%)]	37 (54.41%)	18 (66.67%)	0.459
	HTN [n(%)]	29 (42.64%)	13 (48.14%)	0.830
Severity at presentation	Moderate [n(%)]	44 (64.7%)	16 (59.25%)	0.432
	Severe [n(%)]	23 (33.8%)	12 (44.44%)	
Other co-medications	HCQ [n(%)]	61 (89.7%)	21 (77.78%)	0.79
	Azithromycin [n(%)]	60 (88.23%)	23 (85.18%)	1.00
	Fevipiravir [n(%)]	00	00	N/A
	LMWH [n(%)]	68 (100%)	27 (100%)	NA
	Dexamethasone [n(%)]	68 (100%)	27 (100%)	NA
Clinical symptoms	Vomiting [n(%)]	6 (8.82%)	1 (3.7%)	0.670
	Nausea [n(%)]	30 (44.11%)	7 (25.92%)	0.063
	Diarrhea [n(%)]	27 (39.7%)	12 (44.44%)	0.861
	SOB [n(%)]	42 (61.76%)	20 (74.07%)	0.466
	Cough [n(%)]	49 (72%)	21 (77.78%)	0.939
	Sore throat [n(%)]	37 (54.4%)	14 (51.85%)	0.590
	Fever [n(%)]	48 (70.58%)	21 (77.78%)	0.820

Efficacy and safety of early initiation (within 9 days) versus late initiation (>9 days) of remdesivir

Early initiation of remdesivir was associated with a significant benefit in terms of mortality and requirement of mechanical ventilation. However, no significant differences were seen in terms of ICU requirement. Statistical adjustments were carried out regarding important prognostic factors and baseline imbalances (age, sex, disease severity, CPT use, and serum creatinine level) using multivariate logistic regression. The beneficial effects of early initiation of remdesivir were maintained for parameters like mortality (adjusted p-value 0.003), and requirement of mechanical ventilation (adjusted p-value 0.003) even after statistical adjustment (Table 4). Late initiation of remdesivir was associated with a significantly higher occurrence of worsening dyspnea and the alteration of liver function (Table 4).

**Table 4:** Comparative efficacy and safety of early-initiation remdesivir (within 9 days of symptom onset) versus late-initiation remdesivir (>9 days) among COVID-19 patients p* indicates adjusted p-value, for which adjustments were made in terms of age, sex, serum creatinine level, disease severity, and CPT use. CPT: convalescent plasma therapy; ALT: alanine aminotransferase

Endpoint		R within 9 days of symptom onset, n=67	R started >9 days of symptom onset, n=28	P value	P*
Mortality		1 (1.49%)	9 (32.14%)	<0.001	0.003
Requirement of mechanical ventilation		1 (1.49%)	11 (39.28%)	<0.001	0.003
Requirement of ICU		29 (43.28%)	18 (64.28%)	0.062	0.119
Side effects	Nausea	35 (52.23%)	15 (53.57%)	0.962	0.898
	Worsening dyspnea	5 (7.46%)	10 (35.7%)	0.001	0.003
	Elevated ALT	2 (2.98%)	7 (25%)	0.002	0.007

## Discussion

In our study, remdesivir alone and remdesivir + CPT groups were similar in terms of baseline characteristics except for the proportion of population with a serum creatinine level >1.5, which was higher in the remdesivir alone group. Similarly, the early and late-initiation of remdesivir groups were also comparable in terms of demographic and baseline characteristics except for the fact that the proportion of patients with serum creatinine level >1.5 was higher in the early-initiation of remdesivir group.

Comparative efficacy and safety of remdesivir versus remdesivir + CPT

In our study, the addition of CPT to remdesivir therapy in moderate and severe COVID-19 patients had no additional benefit in terms of survival and requirement of mechanical ventilation and ICU compared to remdesivir therapy alone. Our findings are also supported by a recent Cochrane Review that included 20 studies (one randomized controlled trial, three interventional non-randomized controlled studies, and 16 interventional non-controlled and non-randomized studies) and concluded that the effectiveness of CPT in decreasing mortality or providing clinical improvement is uncertain in patients with COVID-19 [31].

Adverse events like nausea, worsening of dyspnea and alteration in *LFTs *were observed among patients in both groups, although no significant differences in them were observed between the two groups.

Comparative efficacy and safety of early-initiated remdesivir versus late-initiated remdesivir

In our study, all moderate and severe COVID-19 patients received remdesivir. It was observed that mortality and the requirement of mechanical ventilation were lower in patients initiating remdesivir therapy within 9 days after onset of their first symptom compared to those in patients initiating remdesivir therapy after over 9 days of the symptom onset. The benefits were evident even after adjustment for major baseline imbalances and major prognostic factors (e.g., age, sex, serum creatinine level, disease severity, and CPT use).

Adverse events like worsening dyspnea and alteration of liver function were higher in the late-initiated remdesivir group. This may be due to a primary effect of the drug or progression of disease and subsequent metabolic alteration and clinical deterioration.

Many studies report the safety and efficacy of remdesivir, e.g., remdesivir versus placebo [22,23], and remdesivir 5 days versus 10 days [27]. However, the timing of initiation of remdesivir is compared in very few studies [28]. Similar to Mehta et al. [28], in our study, the early initiation of remdesivir was also associated with a significant mortality benefit. A previous study among adult patients with severe Covid-19 by Wang et at. had observed no significant clinical benefit with remdesivir therapy as compared to placebo. However, it was found that patients receiving remdesivir within 10 days of symptoms onset had a faster clinical recovery rate [22]. In a recent study among predominantly non-white hospitalized patients with COVID-19, it was observed that use of remdesivir was associated with shorter time to clinical improvement (median 5 days vs 7 days) and decreased 28 days mortality rate (7.7% vs 14%) as compared to matched controls who didn't receive remdesivir; although the difference was clinically insignificant. The addition of corticosteroids to remdesivir did not reduce the time to death at 28 days compared with patients who were not administered steroids [32]. In a further analysis, pooled data from all zones of India would give a more strong message on the effectiveness of our study protocol of early use of remdesivir in Covid-19 patients to prevent morbidity and mortality.

Limitations

This was a hospital-based study with a small sample size and was conducted over a limited period of time. Also, the effect of remdesivir on viral load in the patients was not measured in our study. Therefore, a broader randomized study covering a larger number of patients over a longer time period is required in order to gather more detailed information regarding the clinical profile and outcome of patients with COVID-19.

## Conclusions

This is an observational study on comparative safety and efficacy of remdesivir versus remdesivir + CPT and the effect of timing of initiation of remdesivir in COVID-19 patients and described the findings of the study compared to the current literature. The early initiation of remdesivir was associated with a clinical benefit in terms of mortality and mechanical ventilation requirement. However, CPT as an additional therapeutic modality to remdesivir was not found to be beneficial. The response of therapy and mortality rate can be greatly influenced by population genetics as well as ethnic background and food habits, which need further validation. This can be helpful in extrapolating data and preventing recurrent waves of the virus surge across the world.

## References

[1] Prajapat M, Sarma P, Shekhar N (2020). Update on the target structures of SARS-CoV-2: a systematic review. Indian J Pharmacol.

[2] The Lancet (2020). Emerging understandings of 2019-nCoV. Lancet.

[3] Sarma P, Kaur H, Kaur H (2020). Ocular manifestations and tear or conjunctival swab PCR positivity for 2019-nCoV in patients with COVID- 19: a systematic review and meta-analysis. SSRN.

[4] Ciaffi J, Meliconi R, Ruscitti P, Berardicurti O, Giacomelli R, Ursini F (2020). Rheumatic manifestations of COVID-19: a systematic review and meta-analysis. BMC Rheumatol.

[5] Clinical Management Protocol: COVID-19 Version 5, Dated 03.07.20. https://www.mohfw.gov.in/pdf/UpdatedClinicalManagementProtocolforCOVID19dated03072020.pdf.

[6] Sun P, Qie S, Liu Z, Ren J, Li K, Xi J (2020). Clinical characteristics of hospitalized patients with SARS-CoV-2 infection: a single arm meta-analysis. J Med Virol.

[7] Paranjpe I, Russak AJ, De Freitas JK (2020). Clinical characteristics of hospitalized Covid-19 patients in New York City. medRxiv : the preprint server for health sciences.

[8] Sarma P, Bhattacharyya A, Kaur H (2020). Efficacy and safety of steroid therapy in COVID-19: a rapid systematic review and meta-analysis. Indian J Pharmacol.

[9] Rezagholizadeh A, Khiali S, Sarbakhsh P, Entezari-Maleki T (2021). Remdesivir for treatment of COVID-19; an updated systematic review and meta-analysis. Eur J Pharmacol.

[10] Sarma P, Kaur H, Kumar H (2020). Virological and clinical cure in COVID-19 patients treated with hydroxychloroquine: a systematic review and meta-analysis. J Med Virol.

[11] Skipper CP, Pastick KA, Engen NW (2020). Hydroxychloroquine in nonhospitalized adults with early COVID-19: a randomized trial. Ann Intern Med.

[12] Bhattacharyya A, Sarma P, Kaur H, Medhi B (2021). Hydroxychloroquine in nonhospitalized adults with early COVID-19. Ann Intern Med.

[13] Prakash A, Singh H, Kaur H (2020). Systematic review and meta-analysis of effectiveness and safety of favipiravir in the management of novel coronavirus (COVID-19) patients. Indian J Pharmacol.

[14] Kaur H, Shekhar N, Sharma S, Sarma P, Prakash A, Medhi B (2021). Ivermectin as a potential drug for treatment of COVID-19: an in-sync review with clinical and computational attributes. Pharmacol Rep.

[15] Agarwal A, Mukherjee A, Kumar G, Chatterjee P, Bhatnagar T, Malhotra P (2020). Convalescent plasma in the management of moderate covid-19 in adults in India: open label phase II multicentre randomised controlled trial (PLACID Trial). BMJ.

[16] Prajapat M, Sarma P, Shekhar N (2020). Drug targets for corona virus: a systematic review. Indian J Pharmacol.

[17] Kaur H, Sarma P, Bhattacharyya A, Prajapat M, Kumar S, Prakash A, Medhi B (2021). Folic acid as placebo in controlled clinical trials of hydroxychloroquine prophylaxis in COVID-19: is it scientifically justifiable?. Med Hypotheses.

[18] Rico-Mesa JS, Rosas D, Ahmadian-Tehrani A, White A, Anderson AS, Chilton R (2020). The role of anticoagulation in COVID-19-induced hypercoagulability. Curr Cardiol Rep.

[19] Wang M, Cao R, Zhang L (2020). Remdesivir and chloroquine effectively inhibit the recently emerged novel coronavirus (2019-nCoV) in vitro. Cell Res.

[20] Commissioner O of the (2021). COVID-19 update: FDA broadens emergency use authorization for Veklury (remdesivir) to include all hospitalized patients for treatment of COVID-19. https://www.fda.gov/news-events/press-announcements/covid-19-update-fda-broadens-emergency-use-authorization-veklury-remdesivir-include-all-hospitalized.

[21] (2021). FDA issues emergency use authorization for convalescent plasma as potential promising COVID-19 treatment, another achievement in administration’s fight against pandemic. https://www.fda.gov/news-events/press-announcements/fda-issues-emergency-use-authorization-convalescent-plasma-potential-promising-covid-19-treatment.

[22] Wang Y, Zhang D, Du G (2020). Remdesivir in adults with severe COVID-19: a randomised, double-blind, placebo-controlled, multicentre trial. Lancet.

[23] Beigel JH, Tomashek KM, Dodd LE (2020). Remdesivir for the treatment of Covid-19 - final report. N Engl J Med.

[24] Spinner CD, Gottlieb RL, Criner GJ (2020). Effect of remdesivir vs standard care on clinical status at 11 days in patients with moderate COVID-19: a randomized clinical trial. JAMA.

[25] Pan H, Peto R, Henao-Restrepo AM (2021). Repurposed antiviral drugs for COVID-19 - interim WHO solidarity trial results. N Engl J Med.

[26] Kalil AC, Patterson TF, Mehta AK (2021). Baricitinib plus remdesivir for hospitalized adults with Covid-19. N Engl J Med.

[27] Goldman JD, Lye DC, Hui DS (2020). Remdesivir for 5 or 10 days in patients with severe Covid-19. N Engl J Med.

[28] Mehta RM, Bansal S, Bysani S, Kalpakam H (2021). A shorter symptom onset to remdesivir treatment (SORT) interval is associated with a lower mortality in moderate-to-severe COVID-19: a real-world analysis. Int J Infect Dis.

[29] Bornstein SR, Rubino F, Khunti K (2020). Practical recommendations for the management of diabetes in patients with COVID-19. Lancet Diabetes Endocrinol.

[30] (2021). Guidelines on clinical management of severe acute respiratory illness (SARI) in suspect/confirmed novel coronavirus (nCoV) cases. https://ncdc.gov.in/WriteReadData/l892s/96997299691580715786.pdf.

[31] Piechotta V, Chai KL, Valk SJ (2020). Convalescent plasma or hyperimmune immunoglobulin for people with COVID-19: a living systematic review. Cochrane Database Syst Rev.

[32] Garibaldi BT, Wang K, Robinson ML (2021). Comparison of time to clinical improvement with vs without remdesivir treatment in hospitalized patients with COVID-19. JAMA Netw Open.

